# Novel Evidence-Based Combination of Plant Extracts with Multitarget Mechanisms of Action for the Elimination of Hot Flashes during Menopause

**DOI:** 10.3390/molecules27041221

**Published:** 2022-02-11

**Authors:** Maria Tsoumani, Panagiota Efstathia Nikolaou, Aikaterini Argyropoulou, Ioulia Tseti, Sofia Mitakou, Ioanna Andreadou

**Affiliations:** 1 Laboratory of Pharmacology, School of Pharmacy, National and Kapodistrian University of Athens, 15771 Athens, Greece; mtsouman@pharm.uoa.gr (M.T.); nayanik@pharm.uoa.gr (P.E.N.); 2 Division of Pharmacognosy and Natural Products Chemistry, School of Pharmacy, National and Kapodistrian University of Athens, 15771 Athens, Greece; katarg@pharm.uoa.gr (A.A.); mitakou@pharm.uoa.gr (S.M.); 3 Intermed S.A., 14564 Athens, Greece; jtsetis@uni-pharma.gr

**Keywords:** hot flashes, menopause, Black cohosh, Evening primrose, *Hypericum perforatum*, *Glycyrrhiza glabra*, nutraceuticals

## Abstract

Hot flashes are considered the most bothersome complaint during menopause. Although hormone therapy is an effective option to relieve hot flashes, it has been associated with significant side effects. The aim of our study is to suggest a novel combination of different plant extracts with distinct mechanisms of action against hot flashes. We selected the rhizome of *Glycyrrhiza glabra* L. (Fabaceae), the rhizome of *Actaea racemosa* L. (Ranunculaceae), the aerial parts of *Hypericum perforatum* L. (Hypericaceae) to produce extracts rich in bioactive phytochemicals and the seed oil of *Oenothera biennis* L. (Onagraceae). We investigated their estrogenic and antioxidant potential and their inhibitory effect against prostaglandin D2 receptor 1 (DP1) as a novel mechanistic pathway for vasodilation in hot flashes, alone or in combination. The phytochemical footprint of the extracts was analyzed using HPLC-PDA and UPLC-HRMS. We observed that the tested extracts possess different mechanisms of action. *A. racemosa* exerts a beneficial activation of the estrogen receptor, *H. perforatum* possesses the highest antioxidant capacity and the seed oil of *O. biennis* inhibits the DP1 receptor. The triple combination in the optimal doses pertains to efficacy against all three mechanisms of action, serves as a multitarget plant-based therapy and could serve as a novel strategy for the alleviation of hot flashes in postmenopausal women.

## 1. Introduction

Menopause is one of the most critical phases in a woman’s life because it marks the final and irreversible termination of the female reproductive life span, a negative reinforcement of the aging process and loss of sexual attractiveness [1]. During this period the decrease in female hormones (estrogen and progesterone) is accompanied by the appearance of physical, mental and vasomotor symptoms (VMS) [2]. The most pronounce symptom of VMS is the episodes of profuse heat accompanied by sweating and flushing, experienced more intensely in the upper torso (head, neck, chest, and upper back), also known as hot flashes [3]. Hot flashes are experienced by approximately 73% of postmenopausal women and 50.3% to 82.1% of menopausal women have reported that hot flashes or night sweats lead to a reduction in their quality of life and increased use of medical resources [4].

Medical treatments for menopausal hot flashes are available and the current gold-standard treatment is estrogens, more specifically, 17β-estradiol. Despite the effectiveness of the estrogens (reduction of hot flashes by 75%), their use has been associated with side effects such as nausea, dizziness and dry mouth. Given the potential negative impact of hormone replacement therapy (HRT) on cardiovascular health, such as the increased risk of venous thromboembolic events, pulmonary embolism and deep vein thrombosis [5], 40% to 50% of women in Western countries who experienced hot flashes chose to use complementary therapies, including plant-based treatments. A broad range of plant-based therapies has been developed to relieve menopausal hot flashes, including soy isoflavones and soy extracts and other medicinal herbs. In the current study, we focused on the plants of *Actaea racemosa* L., also known as Black Cohosh (BC), *Oenothera biennis* L., also known as Evening Primrose (EP), *Glycyrrhiza glabra* L. (GG) or Liquorice and *Hypericum perforatum* L. (HP) or St. John’s Wort, which are rich in bioactive polyphenols, including flavones and lignans that mimic the functions of endogenous estrogens [6]. Notably, these plants have been studied for treatment of menopausal symptoms [6]; however, there is insufficient scientific data on their effect specifically on their underlying mechanisms of action. Cutaneous vasodilation in the skin is a common peripheral vascular event that mediates hot flashes during menopause. The exact mechanism of cutaneous vasodilation is unknown; however, nitric oxide- (NO) or prostaglandin-dependent mechanisms have been described to contribute to this neuronally-mediated vasodilatory response [7,8]. In addition, oxidative stress is involved in the pathogenesis of hot flashes. The repeated episodes of hot flashes contribute to oxidative stress production by raising the level of reactive oxidant species and by blocking antioxidants and their function in neutralizing reactive oxygen/nitrogen species [9].

The aim of this study was to test the hypothesis that BC, HP, GG and EP extracts that are enriched in bioactive ingredients could alleviate the menopausal hot flashes episodes. Therefore, we examined their estrogenic effect, their antioxidant activity, as well as their impact on blocking the prostaglandin-induced vasodilation as possible mechanisms to alleviate hot flashes. The overall target of the study is to design the optimal extract combination consisting of different plant species with distinct mechanisms of action against hot flashes.

## 2. Results

### 2.1. Production of Extracts and Identification of their Phytochemical Footprint

The selected plants were extracted using the exhaustive and efficient ultrasound assisted extraction technique with two different solvent systems (water and water/methanol). In order to differentiate the extracts of each plant based on the solvent used to produce the extracts, we used the suffix “a” for the aqueous extracts and “b” for the water/methanol extracts. For the phytochemical profiling of the extracts, HPLC-PDA and UPLC-HRMS were employed. The extracts showed a particularly rich chemical profile in the HPLC-PDA analysis, mainly at wavelengths of 254 and 280 nm, where characteristic groups of bioactive substances absorb. The HPLC chromatograms of the extracts of HP, BC and GG are presented in Supplementary Figures S1–S3, showing that the use of solvents of different polarity caused a minor impact in the compounds extracted from the same plant and that the most important absorption peaks were similar. Further identification of the major metabolites was performed by LC-HRMS analysis; difference in the main metabolites of the two extracts of BC with different polarity was observed.

Analysis of HP extracts showed that they were rich in secondary metabolites, with the main metabolites being caffeoylquinic Acid I (CQA), hyperoside and quercetin. Other characteristic substances of HP belonged to the categories naphthodianthrones and phloroglucinols (hyperforin, pseudohypericin and hypericin) [10] were found to a lesser extent (Supplementary Tables S1 and S2).

According to LC-HRMS analysis, extracts of BC contain a variety of substances. More specifically, the aqueous extract (BCa) was dominated by caffeic acid derivatives, cimicifugic acid E/F and cimiracemate A/B, while, in the case of the aqueous/alcoholic (BCb) extract, the main ones were the substances fukinolic acid, cimicifugic acid A/B, cimicifugic acid E/F and cimiracemate A/B (Supplementary Tables S3 and S4). The chemical profile of both extracts coincides with the literature data on the chemical composition of BC [11].

Regarding the GG extracts, the substances p-hydroxybenzylmalonic acid, liquiritin apioside and glycyrrhizic acid were the main metabolites, without any differences between the two different polarity extracts (Supplementary Tables S5 and S6). Both extracts contained substances characteristic of GG such as flavonoids, flavanones, isoflavones and flavonoids (glabridin, glabrone, glabrol, 3-hydroxyglabrol, glabrene, glycybridin J, glycybridin C, licoflavone C, kanzonol A, kanzonol Y), as well as saponins (licorice saponin G2, licorice saponin B2, licorice saponin K2/H2) [12,13].

In the case of EP extract, its constituents were identified by LC-MS analysis after methylation (Supplementary Table S7). In agreement with literature data [14], it was found that the sample contained a high content in polyunsaturated fatty acids and that, specifically, γ-linolenic acid was the main compound.

### 2.2. Estrogenic Effect of the Extracts on the MCF-7 Cells

**Figure 1:**
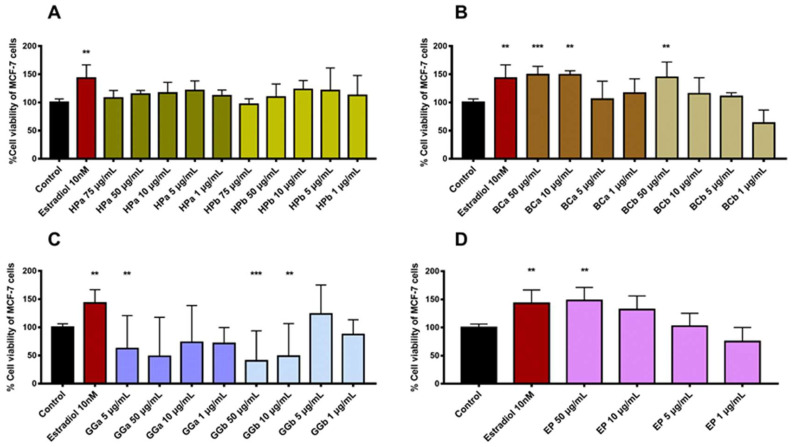
Comparison of the estrogenic effects of (**A**) HPa and HPb, (**B**) BCa and BCb, (**C**) GGa and GGb and (**D**) EP extracts on MCF-7 cell proliferation assay measured by MTT. The bar graph depicts mean± SD of 3 different biological replicates performed in triplicates; the data were analyzed with 1-way ANOVA, followed by Dunnet’s multiple comparison test. Statistical symbols indicate significance in comparison to the control group (** *p* < 0.01, *** *p* < 0.001).

In the present study, we evaluated the estrogenic effect of the extracts on the estrogen-response (ER)-positive MCF-7 cell line and then defined the ER-like activity on the combinations of the most active extracts. The estrogenic activity was determined as the increase of MCF-7 viability and was significantly increased when the cells were treated with BCa 50 μg/mL (*** *p* < 0.001), BCa 10μg/mL (** *p* < 0.01), BCb 50 μg/mL (** *p* < 0.01) and EP 50 μg/mL (** *p* < 0.01). In parallel, the extracts of GG, namely GGa and GGb exhibited an inhibitory effect on the MCF-7 growth indicating a possible anti-proliferative effect (Figure 1). Therefore, taking our results together, the produced extracts are rich in bioactive compounds and the combination therapy against hot flashes should include BCa 50μg/mL, BCa 10μg/mL, BCb 50μg/mL or EP 50μg/mL extract to possess the estrogenic effect.

### 2.3. Antioxidant Capacity of the Tested Extracts

2,2-diphenyl-1-picrylhydrazyl (DPPH) is considered as an accurate methodology for the evaluation of the antioxidant capacity of extracts and/or pure compounds, widely used in the field of natural products; it is expressed as percentage (%) of oxidative inhibition [15]. Our results suggest that HP and BC exhibit antioxidant effects. Comparing the water and water/methanol extracts from each plant, we found that water/methanol extracts were more effective. Importantly, the water/methanol extract of HP (HPb) possessed the most effective antioxidant capacity against DPPH, with an IC_50_ value of 74.7 ± 1.4 μg/mL, whereas the IC_50_ value for the BCb was calculated to 129.5 ± 1.5 μg/mL. Additionally, the water extract of HP (HPa) exhibited remarkable antioxidant activity against DPPH, with an IC_50_ value of 148.4 ± 1.0 μg/mL. The IC_50_ values for all the tested extracts are depicted in Table 1 and the graphs reporting the dose response biological activity are demonstrated in Supplementary Figures S4 and S5. Our results confirm that the extracts are enriched with secondary plant metabolites with antioxidant efficiency and that the HPb extract is the most effective one. It must be noted that EP oil extract showed no antioxidant activity against DPPH and that, therefore, the IC_50_ value could not be calculated. Based on our results, we deduced that the novel effective combination should contain the extracts HPb, or BCa to pertain antioxidant effect.

### 2.4. Inhibitory Effect of the Extracts on Prostaglandin Induced Vasodilation

**Figure 2:**
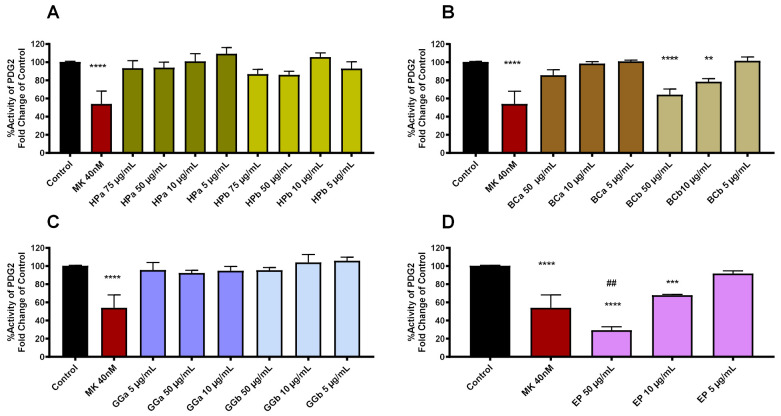
Activity of DP1 receptor based on the downstream measurement of cAMP production. Data are produced by the comparison of cAMP production between the control and the positive control MK-0524 with the extracts (**A**) HPa and HPb, (**B**) BCa and BCb, (**C**) GGa and GGb and (**D**) EP. The bar graph depicts mean± SD of three different biological replicates (n = 3 healthy volunteers) performed in duplicates; the data were analyzed with 1-way ANOVA, followed by Dunnet’s multiple comparison test. Statistical symbols indicate significance in comparison to the control group (* *p* < 0.05, ** *p* < 0.01, *** *p* < 0.001, **** *p* < 0.0001) and in comparison, to the positive control MK-0524 inhibitor (## *p* < 0.01).

Currently, the data on the mechanism of cutaneous vasodilation during the hot flashes are scarce. For the mechanism of vasodilation, previous studies have shown that ~85–95% of the increase in blood flow to the skin is caused by an active vasodilator system in which prostaglandins and NO play an important role [16]. Herein, we examined the inhibitory effect of the seven produced extracts on the prostaglandin D2 receptor 1 (DP1) as a novel mechanistic pathway in combination with others to combat hot flashes in menopause Our results demonstrate that EP extract (50 μg/mL and 10μg/mL, **** *p* < 0.0001 and ** *p* < 0.01 respectively) and BCb (50 μg/mL and 10 μg/mL, **** *p* < 0.0001 and ** *p* < 0.01 respectively) reduce cAMP production of platelet rich plasma (PRP) in a dose-dependent manner, indicating a dose-dependent inhibitory effect on the DP1 receptor (Figure 2). The biological activity of the BCb extract at 50 μg/mL was similar to the positive control (P = NS in comparison to the MK-0524 inhibitor at 40 nM) and EP extract at 50 μg/mL, which demonstrates an enhanced inhibitory effect compared to the MK-0524 inhibitor (## *p* < 0.01 compared to MK-0524 group). Therefore, EP 50 μg/mL should be used as an essential ingredient in the combination therapy, which will result in the reduction in prostaglandin D2 (PGD2) secretion into the skin, leading to vasodilation.

### 2.5. Evaluation of Extract Combinations on the Different Biological Assays

**Figure 3:**
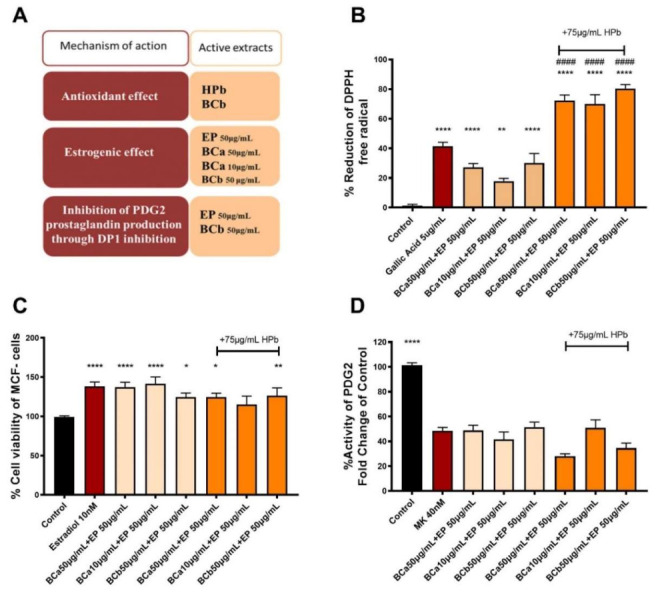
(**A**) Diagram demonstrating the most active extracts as a monotherapy per mechanism of action. The plots depict the effect of the six different combinations of extracts on (**Β**) DPPH radical scavenging activity, (**C**) MCF-7 cell proliferation assay measured by MTT and (**D**) the activity of DP1 receptor by cAMP measurement. The bar graphs depict mean± SD of three different biological replicates. The data were analyzed with 1-way ANOVA, followed by Dunnet’s multiple comparison tests. Statistical symbols indicate significance in comparison to the control group (* *p* < 0.05, ** *p* < 0.01, **** *p* < 0.0001) or the positive control of the assay (^####^ *p* < 0.0001).

Considering the results from the investigation of the extracts on three different mechanisms of action (Figure 3A), we subsequently evaluated six different combinations in order to identify the optimal extract combination. Based on the estrogenic and DP1 activity assay, we chose three double combinations containing BC extract in 50 μg/mL, 10 μg/mL or 50 μg/mL dose combined with EP at the dose of 50 μg/mL. For the triple combination therapy, we added HPb at the concentration of 75 μg/mL to ensure that the combination exerts significant antioxidant activity. Of note, since the GG extracts did not exhibit biological activity in the investigated mechanisms, they were excluded from the rationale of the combinations a priori.

As far as the antioxidant effect of the combinations is concerned, our results demonstrated that the double combinations were less effective compared to the positive control of the assay, gallic acid (Figure 3B). The double combination containing BCa 10 μg/mL was less effective than the combinations containing BCa or BCb at the concentration of 50 μg/mL, indicating a dose response effect. The addition of the HPb extract in the double combinations at the concentration of 75 μg/mL resulted in the creation of triple combinations with a remarkable increase of the antioxidant capacity against DPPH (**** *p* < 0.0001 in comparison to the control group). This addition also led to a significant increase of the radical scavenging activity in comparison to gallic acid (^####^ *p* < 0.0001), indicating a strong antioxidant potential of the triple combination independently of their composition in BCa or BCb. 

The estrogenic assay demonstrated that the double combinations containing BCa and EP were effective in producing estrogen-like activity (**** *p* < 0.0001 in comparison to control). Interestingly, the addition of HPb 75 μg/mL, which did not significantly increase the survival of MCF-7 cells as a monotherapy, slightly reduced the estrogenic potential of the double combinations of BCa and EP (** *p* < 0.01 compared to the control group). This reduction could be of significant value for the translation to the clinical use of the combination, where it could protect from the over-activation of the estrogen pathway (Figure 3C). Finally, the double and the triple combinations were tested as potent inhibitors of the DP1 receptor by measuring cAMP on PRP of healthy volunteers. Our results indicated that all combinations significantly reduced cAMP production in comparison to the control group (**** *p* < 0.0001 control vs. all other groups) which indicates an inhibitory effect on the DP1 receptor (Figure 3D). Therefore, the BCa or BCb with EP alone or in combination with HPb exerted an inhibitory effect on the DP1 receptor and could reduce excessive vasodilation occurring during hot flashes.

## 3. Discussion

For relieving the VMS such as hot flashes during the menopause period, the U.S. Food and Drug Administration (FDA) suggests that estrogen-based therapy is the most effective strategy. However, global data shows that HRT use drops year by year and has caused more detrimental effects than a beneficial impact on menopausal disorders according to the publication of the first results of the Women’s Health Initiative (WHI) [17]. Therefore, there is a large unmet need for finding effective alternatives for treating hot flashes during menopause [18].

Among them, nutraceuticals have drawn public attention because of their possible ability to alleviate menopausal symptoms [19]. The majority of nutraceuticals tested for the alleviation of hot flashes showed inconsistent results in clinical studies [20] unmasking the need for further basic research studies.

The aim of our study was to examine four different plant species rich in bioactive compounds and different extraction procedures to produce different bioactive extracts that could be used alone or in combination against different pathophysiological mechanisms of hot flashes. We investigated two previously described pathophysiological mechanisms responsible for hot flashes, namely, the activation of the estrogen receptor and the oxidative stress, and one novel mechanism, namely, the inhibition of the DP1 receptor, which can alleviate the vasodilatory impact mediated by prostaglandins [21]. Our results revealed that a triple combination of HP, BC and EP is ideal for multi-target therapy against the major climacteric symptom of hot flashes and that each extract is necessary in the combination to provoke the biological response in the distinct mechanisms.

One of the first logical targets for the treatment of menopausal hot flashes would be to stimulate the estrogen receptor. Developing an alternative to HRT without increasing the risk of breast cancer is very important for improving the quality of life of postmenopausal women. Thus, the use of plant-derived active molecules with a scientifically detailed mechanism of action highlights a real need of modern therapy [22]. Our results, using the MCF-7 cell line, showed that BC and EP extracts caused estrogenic effects. Over the last decade, BC has received significant attention as a natural product for menopausal symptom relief. However, the exact mechanism of action is unknown. Many studies have indicated that BC acts as a selective estrogen receptor modulator (SERM), thus, inducing inhibitory growth effects on hormone-dependent cancer cells [23]. However, the results regarding the proliferation of the MCF-7 cells are inconsistent through different studies, since some studies have shown stimulation and others inhibition of MCF-7 proliferation [24,25,26]. This discrepancy could be attributed to the area and the time of collection of the plant raw material, the extraction procedure, the chemical composition of the extract and the treatment doses, which may significantly affect the results. Our tested BC extracts are dominated by cimicifugic acid E/F and cimiracemate A/B, which are considered along with other terpene glycosides to bind and stimulate the estrogen receptor [19]. In fact, a molecular docking study showed that phenolic compounds, such as cimicifugic acid A, cimicifugic acid B, cimicifugic acid G, cimiciphenol, cimiciphenone, cimiracemate A, cimiracemate B, cimiracemate C, cimiracemate D, and fukinolic acid have remarkable docking affinities for both ERα and Erβ receptors [27]. 

Regarding the EP oil extract, to our knowledge, this is the first study indicating that this extract possesses an estrogenic effect. Herein, we report that, when we added HP extract in the double combination of BC and EP, we observed a 25% reduction in the estrogenic effect, which remained significantly increased in comparison to vehicle-treated MCF-7 cells. This observation can be attributed to the fact that HP secondary metabolites, such as hypericin, have been proposed to induce cell death in this cell line [28]. The combination of BC and EP with HP extracts seems to produce a fine-tuning regulation of the estrogen receptor stimulation. 

The second mechanism that we investigated was oxidative stress. Mechanistically, estrogen deficiency along with the physical, psychological, and metabolic complications accompanying hypoestrogenism have been reported to increase oxidative stress [9]. In addition, a recent cross sectional clinical study in postmenopausal women demonstrated that the intensity of hot flashes is associated with oxidative stress, an association which was not evident in premenopausal women [29]. Our study demonstrated that the water/methanol extract of HP (HPb) is an effective antioxidant as a monotherapy and is the most essential ingredient of the triple combination. Our results are consistent with a previous study suggesting that the antioxidant mechanism of HP could be attributed to its free radical scavenging activity, metal-chelation activity, and reactive oxygen quenching activity due to its content in quercetin [30]. In parallel, the BCb extract showed remarkable antioxidant activity since it contains the previously-described antioxidants fukinolic acid, cimicifugic acid A and cimicifugic acid B [31].

Subsequently, we examined the potential effect of the plant extracts in inhibiting DP1 receptors. Inhibiting DP1 receptors would reduce intracellular release of cAMP and lead to decreased vasodilation [21]. Hot flashes in menopause occur following vasodilation of small capillaries under the skin, a response that can be mediated via histamine/bradykinin or prostaglandins. In fact, the activation of G protein-coupled receptor 109A (GPR109A) in dermal Langerhans cells increases the arachidonic acid and the prostaglandins, such as PGD2 and prostaglandin E2 (PGE2), subsequently activating the DP1 receptor, prostaglandin E2 receptor (EP2) and prostaglandin E receptor 4 (EP4) causing cutaneous vasodilation. Thus, inhibiting DP1 receptors can inhibit flushing. Our results have shown, for the first time that EP oil extract has a remarkable inhibitory effect on DP1 receptor. The EP oil obtained from the seeds of *O. biennis* is a rich source of γ-linolenic acid popularly believed to suppress menopausal flashing [32]. Additionally, it is recognized as a potential source of unsaturated fatty acids, such as α-linolenic acid [14], that can block prostaglandin production. In our study, we also observed that the BCb extract reduced cAMP production, indicating inhibition of the DP1 prostaglandin receptor. BC pharmacologically-active components possess estrogenic dopaminergic, serotonergic, and progestogenic effects [32]. Although the clinical effectiveness and tolerability of BC for treating menopausal symptoms has not been proved in randomized clinical trials, we revealed that the BC extract at the dose of 50 μg/mL could reduce excessive vasodilation through the PGD2 receptor. Notably, the triple combination of EP, BC and HP retained the same biological activity, even if HP was ineffective in this mechanism of action, indicating that HP extract did not interfere with the signaling of prostaglandins. Regarding the correlation between DP1 receptor inhibition and the regulation of blood pressure, previous reports in rats indicated that PDG2 administration and DP1 agonism induced blood pressure elevation, but, chronically, the same axis led to the inhibition of the sympathetic system and to the downregulation of blood pressure [33]. Since EP and IP receptors are the main regulators of blood pressure via prostaglandins through control of vascular tone, sodium excretion, and renin release, and not the DP1 [34], we considered that the proposed extract combination is unlikely to influence blood pressure or to cause hypertension. 

GG extracts did not exhibit biological activities in the investigated pathophysiological mechanisms of hot flashes therefore, we did not include GG extracts in the combination experiments. Specifically, we observed that GG extracts did not exhibit an estrogen-like activity, exerted an inhibitory effect on the MCF-7 growth, presented a weak antioxidant efficacy, as it is shown from the relatively high IC_50_ values in the DPPH activity assay and had no effect on the DP1 receptor. Our results on the estrogen-like activity are not consistent with several reports in the literature, which found that Glycyrrhiza extracts and more specifically extracts rich in liquiritigenin mitigated hot flashes symptoms in menopause due to estrogenic effects [6]. These differences can be attributed to the different species of Glycyrrhiza used in the study. In a recent report evaluating the estrogenic efficacy of three different Glycyrrhiza species, *G. glabra, G. uralensis* and *G. inflata,* it was observed that *G. inflata* possessed the most potent estrogenic properties [35]. 

Our study revealed that the triple combination of HPb, BCa or BCb and EP extracts possessed estrogen-like activity, high antioxidant potential and the ability to inhibit the prostaglandin-induced vasodilation and could serve as a multitarget therapy against hot flashes. However, we acknowledge that the over-activation of the estrogen pathway would be a limitation for long-term usage of the extracts. The addition of the HPb extract reduced the estrogenic potential of BCa and BCb extracts in combination with EP; this reduction could be of significant value for the translation to the clinical use of the triple combination.

## 4. Materials and Methods

### 4.1. Reagents 

Extraction solvents were of analytical grade and purchased from Carlo Erba (Milan, Italy). HPLC-grade solvents were supplied by Fisher Scientific (Loughborough, UK). LC–MS grade solvents were purchased from Merck Chemicals (Darmstadt, Germany) and high purity water was provided by a Millipore Direct-Q^®^ 3 UV purification system (Merck Millipore, Darmstadt, Germany). Gallic acid, 2,2-diphenyl-1-picrylhydrazyl (DPPH, 95% purity) and all the cell culture consumables and reagents were purchased from Sigma-Aldrich (St. Louis, MO, USA) unless otherwise stated. 

### 4.2. Plant Materials and Extraction Process

Plant materials of three different plant species were purchased from certified suppliers: the rhizome of *G. glabra*, the rhizome of *A. racemosa* and the aerial parts of *H. perforatum* (*Hypericaceae*). Plant materials were treated according to predefined conditions [36]. Each material was extracted with water (100%) and water/methanol (50:50). For the evaluation of the biological activities of *O. biennis*, we used a commercial product (seed oil) from Nutra Green Biotechnology Co, Ltd, Shanghai 200120, China. 

### 4.3. UPLC-HRMS Analysis of Plant Extracts BC, HP and GG

UPLC-HRMS analysis was performed on an AQUITY system (Waters) connected to a LTQ-OrbitrapR XL hybrid mass spectrometer (Thermo Scientific) as previously described [36]. 

### 4.4. High-Performance Liquid Chromatography–Photodiode Array Detection (HPLC–PDA) Analysis of Plant Extracts BC, HP and GG

The HPLC analysis was performed as previously described [13].

### 4.5. Analysis of the Commercial EP Seed Oil

The fatty acid composition was determined by liquid chromatography following methylation. One hundred milligrams of the sample were incubated for 2 h at 65 °C with twenty milliliters of toluene and 1% H_2_SO_4_ in methanol (*v*/*v*). Next, the reaction mixture was cooled and 60 mL of 5% NaCl were added. The solution was then washed with n-hexane, and, in continuation, the n-hexane phase was washed with 2% NaHCO_3_. The n-hexane phase was dried over anhydrous sodium sulphate and the solvent was then evaporated in a nitrogen stream without heating and was used for analysis. The fatty acid methyl esters were analyzed on an Acquity UPLC system (Waters). Detection was performed on a Sciex TripleTOF^®^ 5600+ mass spectrometer equipped with a DuoSpray™ ion source operated in the positive and negative ESI mode. Separation was achieved on a Fortis Speedcore C18 column (100 × 2.1 mm i.d, 2.6 μm particle size). A UPLC separation gradient was developed in order to efficiently resolve all compounds for a qualitative analysis. The flow rate was set at 0.4 mL/ min and the solvent system was (A) water 0.1% formic acid and (B) acetonitrile. The elution program was: 5% B for 2 min; 100% B in 15 min; and hold for 2 min. After a return to 5% B in 1 min, column equilibration was performed for 4 min at the end of the run. The injection volume was set to 10 μL and samples were injected at 0.4 mg/mL in water-acetonitrile solution (1:1). Spectrometric features were used for identification of the constituents, such as accurate m/z, proposed elemental composition (EC), and ring double bond equivalent (RDBeq) values. The raw data were acquired and processed with Analyst 1.7.1 software from Sciex.

### 4.6. Evaluation of the Estrogenic Effect of the Extracts on MCF-7 Cells

In order to evaluate the estrogenic effect of the extracts, we used the MCF-7 carcinoma cell line, the most widely used cell line with estrogen receptor properties. MCF-7 cells were obtained from the American Type Culture Collection (ATCC; Manassas, VA) and were grown in DMEM high glucose medium supplemented with 10% fetal bovine serum (FBS) and 1% (*v*/*v*) streptomycin/penicillin at 37 °C and 5% CO_2_. The cells were re-cultured every 3–4 days; when they reached 75% confluence, they were seeded in a 96-well plate at 2 × 103 cells/well. After 24 h, the cells were treated with the extracts or the combination of extracts for 72 h (Supplementary Figure S6A). The final concentration of dimethyl sulfoxide (DMSO) in the treated wells did not exceed 1%. 17-β-estradiol was used as a positive control for the assay after evaluating its effective concentration (EC_50_ value) in preliminary experiments at 40 nM (Supplementary Figure S6B). At the end of the protocol, cell viability was assessed using the 3-(4,5-Dimethylthiazol-2-yl)-2,5-diphenyltetrazolium bromide (MTT) assay. The formula that provided the viability is the following:

(1) $$Cell\;viability\;\left(\%\right)=\frac{Abs\;Sample-Abs\;Background}{Average\;of\;\left(Abs\;Control-Abs\;Background\right)\;}\times 100$$

#### DPPH Radical Scavenging Assay

Free radical scavenging ability of the extracts was tested by DPPH radical scavenging assay, as described before [37,38,39], with some modifications. The hydrogen atom donating ability of the plant extracts was determined by the decolorization of methanol solution of DPPH. All the water/methanol extracts were diluted in DMSO, providing ten different concentrations of 1000 μg/mL, 500 μg/mL, 100 μg/mL, 50 μg/mL, 10 μg/mL, 5 μg/mL, 1 μg/mL, 0.5 μg/mL, 0.1 μg/mL and 0.05 μg/mL, while, for the water extracts, the highest concentration tested was the 100 μg/mL due to low solubility. Gallic acid (5 μg/mL) was used as positive control. All the experiments were conducted twice in triplicates. Percentage DPPH radical scavenging activity was calculated by the following equation:

(2) $$\%DPPH\;radical\;scavenging\;activity=\frac{1-\left(Abs\;sample-Abs\;blank\right)}{Abs0}\;\times \;100$$

where *Abs*0 is the absorbance of the negative control; *Abs sample* is the absorbance of the extracts/standard; *Abs blank* symbolizes the absorbance of samples with EtOH. Then, the % of inhibition was plotted against concentration and IC_50_ values were calculated. 

### 4.7. Determination of the Effect of Extracts against Prostaglandin DP1 Receptor Inhibition

The investigation of the effect of the extracts on DP1 inhibition was performed as previously described [40,41], with slight modifications. Briefly, citrated blood samples from healthy volunteers (*n* = 3) were centrifuged for 15 min at 200× *g* (RT) and PRP was collected. The remaining volume was centrifuged at 1500× *g* for 15 min at RT and the platelet poor plasma (PPP) was collected. Platelets were adjusted to 300,000 platelets/μL with autologous PPP. Isobutylmethylxanthine (IBMX; 0.5 mM final concentration) was added to PRP for 3 min at 37 °C in order to prevent degradation of cyclic adenosine monophosphate (cAMP). Samples (100 μL) of PRP were then pre-incubated (10 min at 37 °C) with various concentrations of extracts in DMSO (2 μg/mL–0.1 μg/mL). In parallel, as positive control of the assay, the previously reported inhibitor MK-0524 was used [40]. Samples were then challenged with PGD2 (600 nM final concentration) added in DMSO and incubated for an additional 10 min at 37 °C. The reaction was then terminated by the addition of 120 μL of HCl 0.1 M to disrupt the cells and to extract the cAMP. The samples were mixed thoroughly and centrifuged at 1400× *g* for 10 min at 4 °C. The supernatant was dissolved in a ratio 1:1 with cAMP assay buffer and used for the ELISA assay. The final concentration of cAMP was determined using a commercially available kit (cAMP kit # ADI—900-067, Enzolife Scientific) based on photometric determination of the amount of extracellular (free cAMP) material in the sample using a reference curve, according to the instructions of the manufacturer. Experiments were performed in three biological replicates using samples from three different volunteers in duplicates. The calculation formula of inhibition percentage was:

(3) $$Inhibition\;\left(\%\right)=\frac{X\;control-X\;sample}{X\;control}\times 100$$

*X control* symbolizes the concentration of cAMP of the control sample without inhibitor, while *X sample* stands for the concentration of cAMP of the tested extracts or positive control.

### 4.8. Statistical Analysis

Visualization of bar graphs and statistical analysis was performed using the GraphPad Prism 7 software (Graph Pad Software, Inc, San Diego, CA, USA). All the results were plotted in graphs as mean ± standard deviation of the mean values. For the evaluation of the DPPH assay results, the IC_50_ values were calculated by transforming the concentration X of the extracts to log10× and by applying nonlinear regression fit to the log (inhibitor) vs. normalized response curve. Comparison among the groups was performed using 1-way ANOVA, followed by Dunnet’s multiple comparison test and the treatments were compared to the respective control. The cut-off for statistical significance was set at *p* < 0.05 (* *p* < 0.05, ** *p* < 0.01, *** *p* < 0.001, # *p* < 0.0001).

## 5. Conclusions

In conclusion, we have discovered a therapeutic intervention that includes three herbal remedies that, in combination, produce the ideal biological response in three distinct mechanisms against hot flashes. Since treating hot flashes with HRT involves several side effects, developing an alternative nutraceutical option is very important for the reduction of VMS. Our triple combination with experimentally documented action on the underlying mechanisms of hot flashes is novel and is expected to lead to an effective improvement in the quality of life of postmenopausal women. Highlighting the therapeutic potential of the extracts can be the basis for creating a nutritional product with strategic placement in emerging markets such as dietary supplements and herbal medicines, which will strengthen society’s confidence in the safety and effectiveness of natural products.

## Figures and Tables

**Table 1:** IC_50_ value of DPPH radical scavenging activity of the different extracts.

Extract Name	DPPH IC_50_ Value
Water extract of HP (HPa)	148.4 ± 1.0 μg/mL
Water/methanol extract of HP (HPb)	74.7 ± 1.4 μg/mL
Water extract of BC (BCa)	274.0 ± 1.0 μg/mL
Water/methanol extract of BC (BCb)	129.5 ± 1.5 μg/mL
Water extract of GG (GGa)	774.7 ± 1.0 μg/mL
Water/methanol extract of GG (GGb)	1059 ± 1.6 μg/mL
Seed oil of EP	No antioxidant effect

## Data Availability

All data and other materials can be obtained from authors.

## Supplementary Materials

Figure S1: HPLC chromatograms of the A. water/methanol and B. Water extract of HP (254 nm—blue, 280 nm—green and 380 nm—purple); Figure S2: HPLC chromatograms of the A. water/methanol and B. water extract of BC (254 nm—blue, 280 nm—green and 380 nm—purple); Figure S3: HPLC chromatograms of the A. water/methanol and B. water extract of GG (254 nm—blue, 280 nm—green and 380 nm—purple); Figure S4: (Left panel) Dose response bar graphs of the DPPH radical scavenging activity and (right panel) IC_50_ graphs of the water extracts of HP, BC, and GG; Figure S5: (Left panel) Dose response bar graphs of the DPPH radical scavenging activity and (right panel) IC_50_ graphs of the water/methanol extracts of HP, BC, and GG; Figure S6: (A) Illustration of the experimental protocol for the investigation of the estrogenic effect on MCF-7 cells (B) The effect of 17-β-estradiol on cell viability of MCF-7 cells. The proliferative effect of 17-β-estradiol on the MCF-7 cells was examined at 7 different concentrations varying from 10,000 nM to 0.01 nM and the EC_50_ value was determined at 42.6 ± 1.8 nM by performing a nonlinear regression fit on the curve log (agonist) vs. response with variable slope (four parameters); Table S1: List of compounds found in the HP water extract (HPa); Table S2: List of compounds found in the HP water/methanol extract (HPb); Table S3: List of compounds found in the water extract of BC; Table S4: List of compounds found in the water/methanol extract of BC; Table S5: List of compounds found in the water extract of GG (GGa); Table S6: List of compounds found in the water/methanol extract of GG (GGb); Table S7: Fatty acid methyl esters composition of the seed oil extract of EP.

## Sample Availability

Samples of the compounds HP, BC, GG and EP are available from the authors.
