# Supplementary material for: Novel Evidence-Based Combination of Plant Extracts with Multitarget Mechanisms of Action for the Elimination of Hot Flashes during Menopause
Source: Molecules. 2022 Feb 11;27(4):1221. doi: 10.3390/molecules27041221 (PMC8874944; doi:10.3390/molecules27041221)
Supplement: Supplementary file 1 [file molecules-27-01221-s001.zip › molecules-1552574-supplementary.pdf]

molecules

MDPI

Article-Supplementary material

Novel evidence-based combination of plant extracts with multitarget mechanisms of action for the elimination of hot flashes during menopause

Maria Tsoumani[1#], Panagiota Efstathia Nikolaou[1#], Aikaterini Argyropoulou[2], Ioulia Tseti[3], Sofia Mitakou[2], Ioanna Andreadou[1,*]

[1]Laboratory of Pharmacology, School of Pharmacy, National and Kapodistrian University of Athens, 15771, Athens, Greece, [2]Division of Pharmacognosy and Natural Products Chemistry, School of Pharmacy, National and Kapodistrian University of Athens, 15771, Athens, Greece, [3]Intermed S.A., Athens, Greece

[#]*These authors have contributed equally to this work and share first authorship*

* Correspondence: jandread@pharm.uoa.gr; Tel.:+302107274827

A.

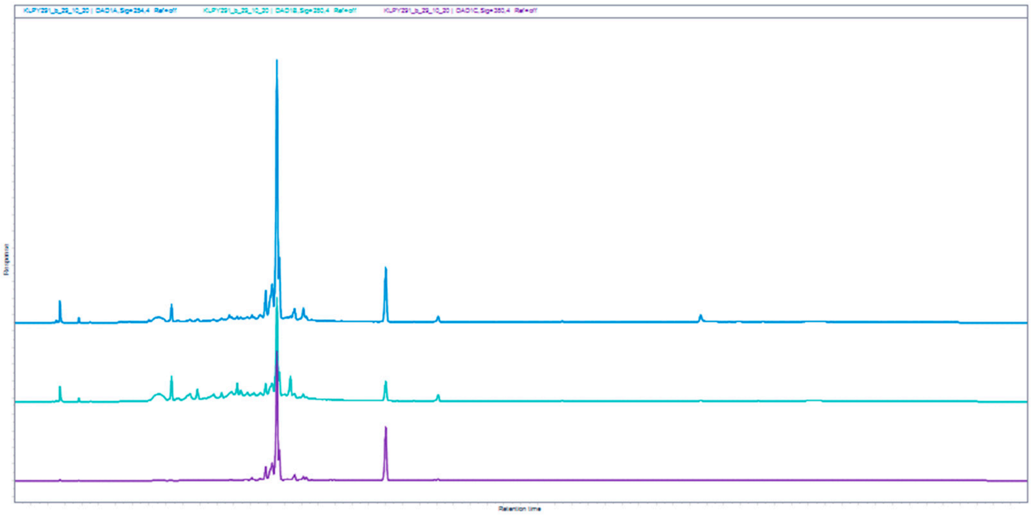

B.

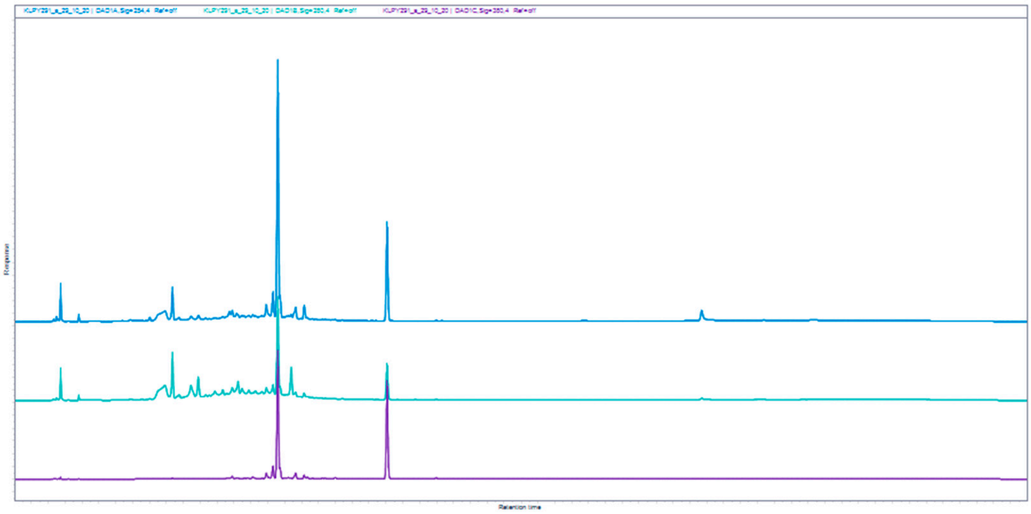

Figure S1. HPLC chromatograms of the A. water/methanol and B. water extract of HP (254 nm – blue, 280 nm – green and 380 nm – purple).

A.

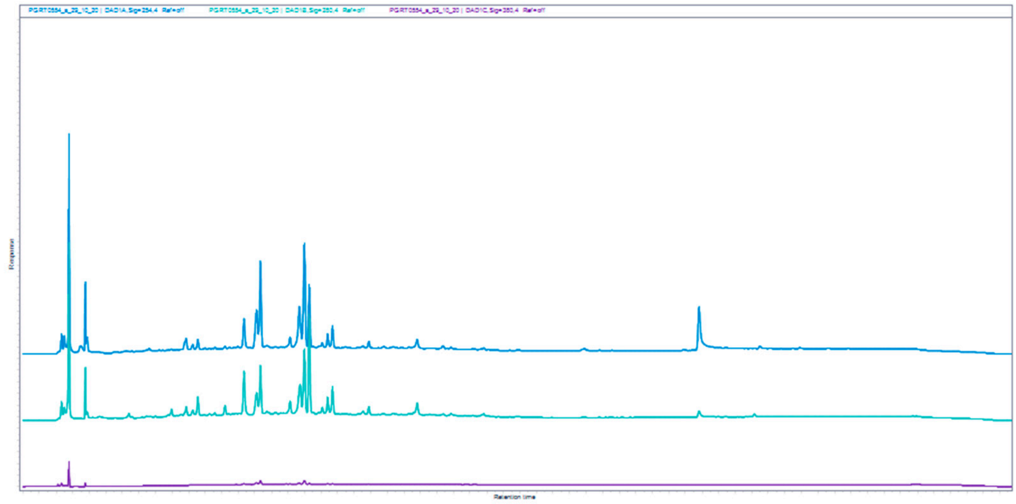

B.

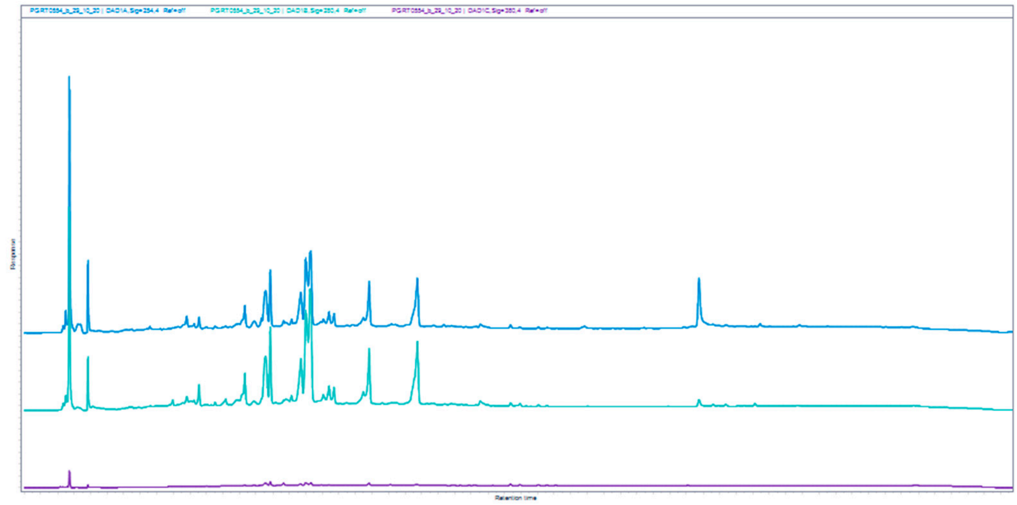

Figure S2. HPLC chromatograms of the **A.** water/methanol and **B**. water extract of BC (254 nm – blue, 280 nm – green and 380 nm – purple).

A.

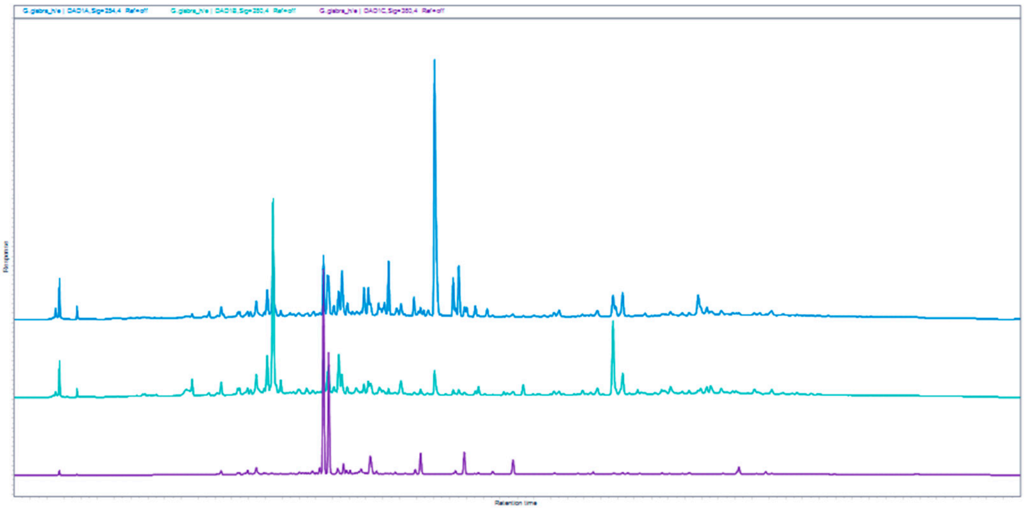

B.

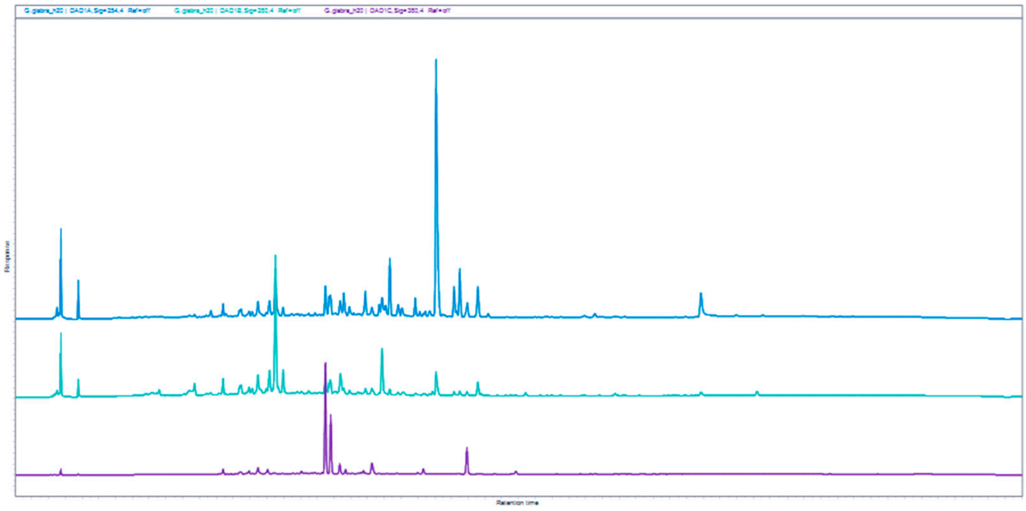

Figure S3. HPLC chromatograms of the **A.** water/methanol and **B.** water extract of GG (254 nm – blue, 280 nm – green and 380 nm – purple).

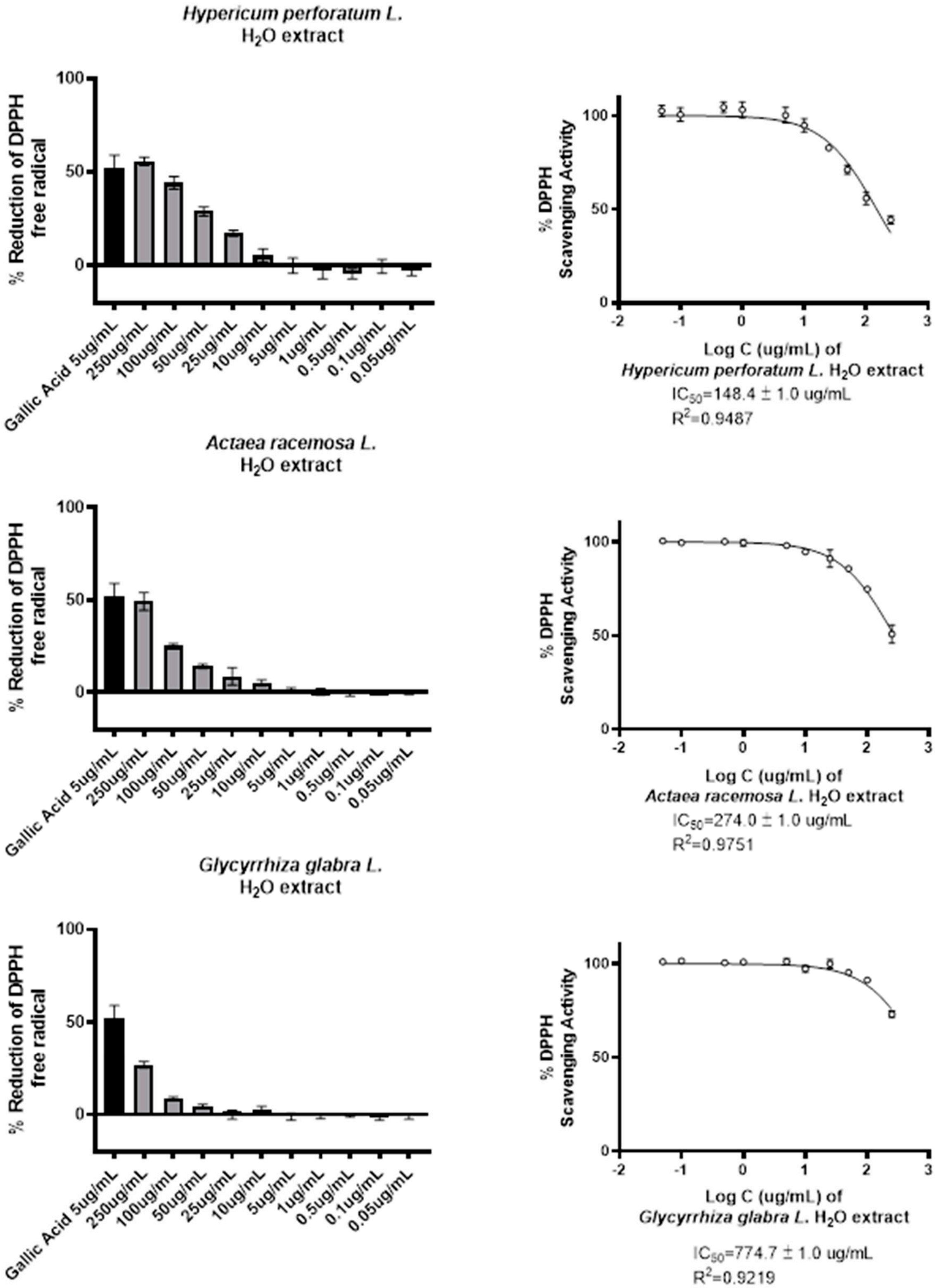

Figure S4: (Left panel) Dose response bar graphs of the DPPH radical scavenging activity and (right panel) IC_{50} graphs of the water extracts of *HP, BC,* and *GG.*

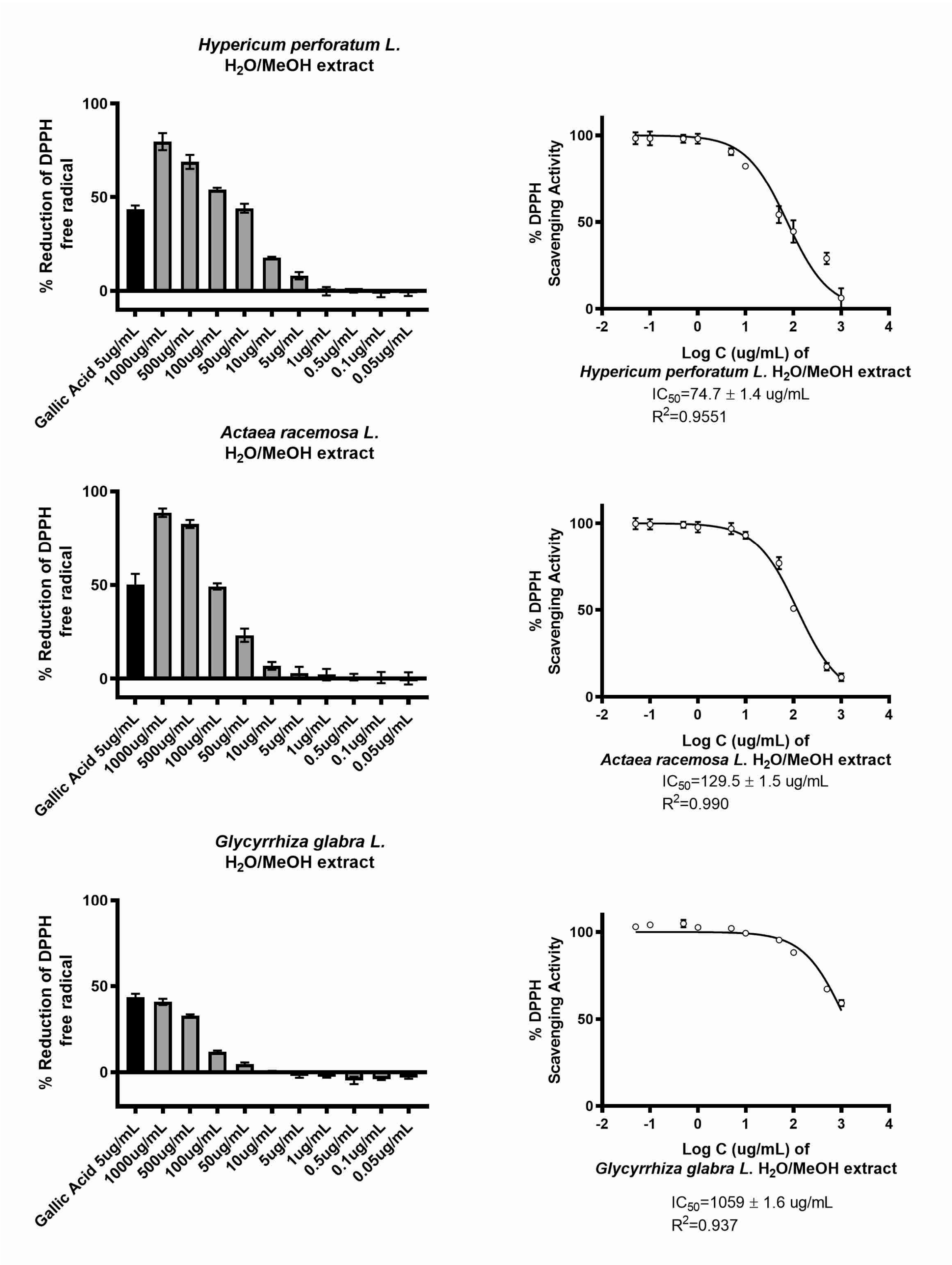

Figure S5: (Left panel) Dose response bar graphs of the DPPH radical scavenging activity and (right panel) IC_{50} graphs of the methanol/water extracts of *HP, BC,* and *GG.*

A

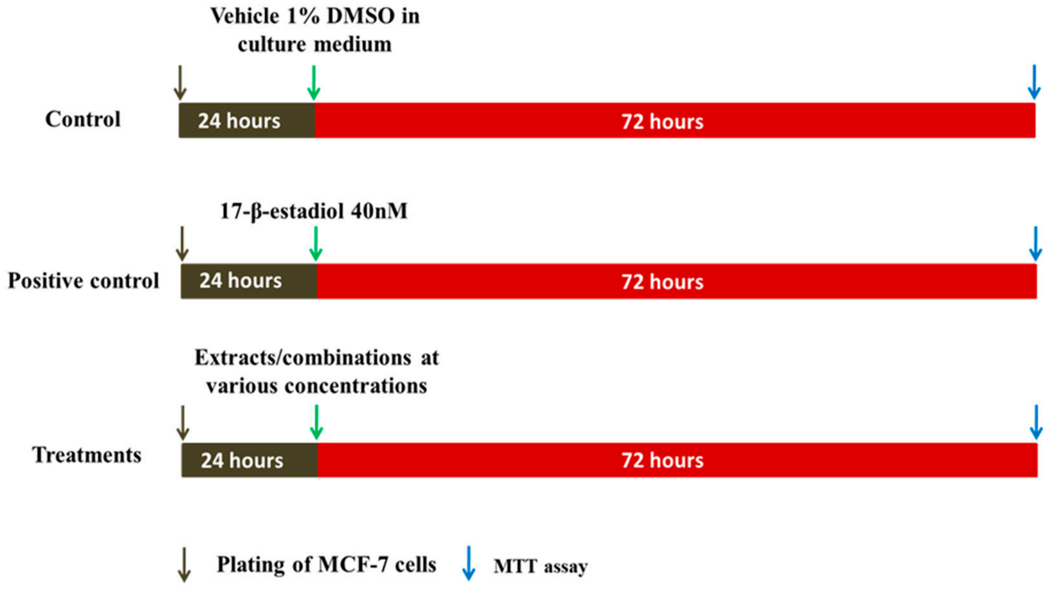

B

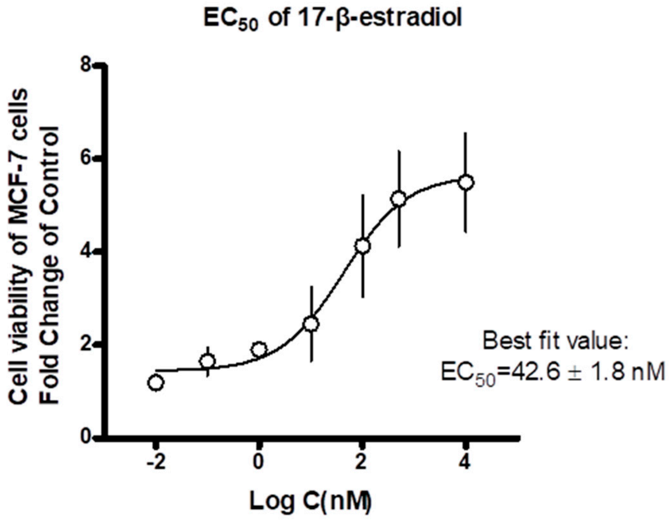

Figure S6. (A) Illustration of the experimental protocol for the investigation of the estrogenic effect on MCF-7 cells (B) The effect of 17-β-estradiol on cell viability of MCF-7 cells. The proliferative effect of 17-β-estradiol on the MCF-7 cells was examined at 7 different concentrations varying from 10000nM to 0.01nM and the EC_{50} value was determined at 42.6±1.8nM by performing a nonlinear regression fit on the curve log(agonist) vs. response with variable slope (four parameters).

Table S1. List of compounds found in the HP water extract (HPa).

			$[M\text{-}H]^-$				
**No**	**Compounds**	**Molecular Formula**	**Rt (min)**	**Theoretical** *m/z*	**Experimental** *m/z*	**Delta (ppm)**	**RDBeq**
1	Caffeoylquinic Acid I (CQA)	$C_{16}H_{18}O_9$	4.81	353.0867	353.0878	-3.13	8.5
2	Flavanol I (Monomeric)	$C_{15}H_{14}O_6$	5.47	289.0723	289.0717	1.86	9.5
3	Caffeoylquinic Acid II (CQA)	$C_{16}H_{18}O_9$	5.49	353.0871	353.0878	-1.99	8.5
4	Chlorogenic Acid (3–CQA)	$C_{16}H_{18}O_9$	5.57	353.0893	353.0878	4.23	8.5
5	Flavanol I (Dimeric)	$C_{30}H_{26}O_{12}$	5.75	577.1374	577.1351	3.90	18.5
6	Flavanol II (Dimeric)	$C_{30}H_{26}O_{12}$	5.76	577.1376	577.1351	4.24	18.5
7	Flavanol II (Monomeric)	$C_{15}H_{14}O_6$	5.84	289.0721	289.0717	1.17	9.5
8	Myricetin hexoside	$C_{21}H_{20}O_{13}$	6.12	479.0851	479.0831	4.14	12.5
9	Quercetin–3–*O*-rutinoside (Rutin)	$C_{27}H_{30}O_{16}$	6.59	609.1489	609.1461	4.58	13.5
10	Quercetin–3–*O*–galactoside (Hyperoside)	$C_{21}H_{20}O_{12}$	6.75	463.0897	463.0882	3.24	12.5
11	Taxifolin 3–*O*–rhamnoside (Astilbin)	$C_{21}H_{22}O_{11}$	6.98	449.1091	449.1089	0.37	11.5
12	Quercetin pentoside	$C_{20}H_{18}O_{11}$	7.10	433.0797	433.0776	4.77	12.5
13	Quercetin 3–acetyl–galactoside	$C_{23}H_{22}O_{13}$	7.12	505.1007	505.0987	3.83	13.5
14	Quercetin 3–*O*–rhamnoside (Quercitrin)	$C_{21}H_{20}O_{11}$	7.25	447.0933	447.0932	0.03	12.5
15	Quercetin–3–*O*–glucoside (Isoquercitrin)	$C_{21}H_{20}O_{12}$	7.41	463.0907	463.0882	5.40	12.5
16	Protohypericin	$C_{30}H_{18}O_8$	7.45	505.0978	505.0928	9.72	22.5
17	Xanthone	$C_{24}H_{20}O_9$	7.80	451.1055	451.1034	4.53	15.5
18	Quercetin	$C_{15}H_{10}O_7$	8.71	301.0354	301.0353	0.08	11.5
19	3,8″–Biapigenin	$C_{30}H_{18}O_{10}$	9.45	537.082	537.0827	-1.34	22.5
20	3′,8″–Biapigenin (Amentoflavone)	$C_{30}H_{18}O_{10}$	9.87	537.0811	537.0815	-2.56	22.5
21	Hyperforin	$C_{35}H_{52}O_4$	19.47	535.3793	535.3855	-2.68	10.5
22	Pseudohypericin	$C_{30}H_{16}O_9$	19.99	519.0742	519.0716	4.99	23.5
23	Hypericin	$C_{30}H_{16}O_8$	20.91	503.0772	503.0772	-0.01	23.5

Table S2. List of compounds found in the HP water/methanol extract (HPb)

			[M-H]-				
No	Compounds	Molecular Formula	Rt (min)	Theoretical	Experimental	Delta (ppm)	RDBeq
				m/z	*m/z*		
1	Caffeoylquinic Acid I (CQA)	$C_{16}H_{18}O_9$	4.89	353.0853	353.0865	-3.56	8.5
2	Empetrifelixin (A or C)	$C_{20}H_{28}O_5$	5.38	347.1841	347.1854	-2.81	7.5
3	Caffeoylquinic Acid II (CQA)	$C_{16}H_{18}O_9$	5.42	353.0889	353.0864	-3.71	8.5
4	Flavanol I (Monomeric)	$C_{15}H_{14}O_6$	5.51	289.0688	289.0709	-3.01	9.5
5	Chlorogenic Acid (3–CQA)	$C_{16}H_{18}O_9$	5.53	353.0881	353.0864	-3.98	8.5
6	Flavanol I (Dimeric)	$C_{30}H_{26}O_{12}$	5.64	577.1319	577.1323	-5.01	13.5
7	Flavanol II (Dimeric)	$C_{30}H_{26}O_{12}$	5.69	577.1346	577.1323	-4.90	18.5
8	Flavanol II (Monomeric)	$C_{15}H_{14}O_6$	5.94	289.0692	289.0710	-2.81	9.5
9	Myricetin hexoside	$C_{21}H_{20}O_{13}$	6.24	479.0812	479.0811	-4.22	12.5
10	Quercetin–3–*O*-rutinoside (Rutin)	$C_{27}H_{30}O_{16}$	6.49	609.1398	609.1431	-4.99	13.5
11	Quercetin–3–*O*–galactoside (Hyperoside)	$C_{21}H_{20}O_{12}$	6.70	463.0852	463.0868	-3.11	12.5
12	Taxifolin 3–*O*–rhamnoside (Astilbin)	$C_{21}H_{22}O_{11}$	6.94	449.1092	449.1074	-3.44	11.5
13	Quercetin pentoside	$C_{20}H_{18}O_{11}$	7.03	433.0731	433.0761	-3.52	12.5
14	Quercetin 3–acetyl–galactoside	$C_{23}H_{22}O_{13}$	7.04	505.0959	505.0968	-3.83	13.5
15	Quercetin 3–*O*–rhamnoside (Quercitrin)	$C_{21}H_{20}O_{11}$	7.20	447.0887	447.0915	-3.95	12.5
16	Quercetin–3–*O*–glucoside (Isoquercitrin)	$C_{21}H_{20}O_{12}$	7.21	463.0844	463.0864	-3.97	12.5
17	Xanthone	$C_{24}H_{20}O_9$	7.85	451.1010	451.1016	-4.07	15.5
18	Quercetin	$C_{15}H_{10}O_7$	8.68	301.0325	301.0347	-2.35	11.5
19	3,8″–Biapigenin	$C_{30}H_{18}O_{10}$	9.60	537.0801	537.0811	-2.98	22.5
20	3′,8″–Biapigenin (Amentoflavone)	$C_{30}H_{18}O_{10}$	9.94	537.0811	537.0814	-2.53	22.5
21	Hyperforin	$C_{35}H_{52}O_4$	19.55	535.3793	535.3852	-2.67	10.5
22	Protopseudohypericin	$C_{30}H_{18}O_9$	19.79	521.0905	521.0870	-1.58	22.5
23	Pseudohypericin	$C_{30}H_{16}O_9$	20.01	519.0738	519.0713	-1.67	23.5
24	Hypericin	$C_{30}H_{16}O_8$	20.94	503.0772	503.0772	-0.01	23.5

Table S3. List of compounds found in the water extract of BC (BCa).

			[M-H]$^-$				
No	Compounds	Molecular Formula	Rt (min)	Theoretical *m/z*	Experimental *m/z*	Delta (ppm)	RDBeq
1	Cimiracemate C/D	$C_{20}H_{20}O_8$	0.89	387.1144	387.1129	-1.70	11.5
2	Fukinolic acid derivative	$C_{11}H_{12}O_8$	4.01	271.0865	271.0912	-0.05	6.5
3	Piscidic acid	$C_{11}H_{12}O_7$	4.54	255.0510	255.0501	-2.50	6.5
4	3,5-dihydroxyphenethanol 3-O-β-D-allopyranoside	$C_{14}H_{20}O_8$	4.63	315.1085	315.1071	-1.44	5.5
5	Bergenin	$C_{14}H_{16}O_9$	5.14	327.0722	327.0708	-0.23	7.5
6	Caffeic acid	$C_9H_8O_4$	5.95	179.0350	179.0346	-2.00	6.5
7	Cimicifugic acid C	$C_{20}H_{18}O_{10}$	7.28	417.0827	417.0808	-2.50	12.5
8	Cimicifugic acid E/F	$C_{21}H_{20}O_{10}$	8.13	431.4792	431.4901	-1.02	12.5
9	Cimiciphenone	$C_{18}H_{16}O_7$	8.25	343.0809	343.0823	-0.86	11.5
10	Cimiracemate A/B	$C_{19}H_{18}O_7$	9.18	357.4789	357.4912	-0.82	11.5

Table S4. List of compounds found in the water/methanol extract of BC (BCb)

			[M-H]$^-$				
No	Compounds	Molecular Formula	t_R (min)	Theoretical *m/z*	Experimental *m/z*	Delta (ppm)	RDBeq
1	Cimiracemate C/D	$C_{20}H_{20}O_8$	0.91	387.1144	387.1131	-0.39	11.5
2	Fukinolic acid derivative	$C_{11}H_{12}O_8$	4.00	271.0854	271.0954	0.28	6.5
3	3,5-dihydroxyphenethanol 3-O-β-D-allopyranoside	$C_{14}H_{20}O_8$	4.63	315.1085	315.1074	-1.74	5.5
4	Bergenin	$C_{14}H_{16}O_9$	5.14	327.0722	327.0708	-0.41	7.5
5	Caffeic acid	$C_9H_8O_4$	5.94	179.0350	179.2689	4.78	6.5
6	Fukinolic acid	$C_{20}H_{18}O_{11}$	6.84	433.7393	433.7244	-0.50	12.5
7	Cimicifugic acid C	$C_{20}H_{18}O_{10}$	7.28	417.0827	417.0811	-1.21	12.5
8	Cimicifugic acid I	$C_{22}H_{22}O_{12}$	7.49	477.1038	477.1019	-1.08	12.5
9	Cimicifugic acid A/B	$C_{21}H_{20}O_{11}$	7.57/7.68	447.0244	447.0232	-1.07	12.5
10	Cimicifugic acid E/F	$C_{21}H_{20}O_{10}$	8.06/8.13	431.0984	431.0967	-1.77	12.5
11	Cimiciphenone	$C_{18}H_{16}O_7$	8.23	343.0809	343.0823	-0.32	11.5
12	Cimiracemate A/B	$C_{19}H_{18}O_7$	9.18	357.4732	357.4329	-0.56	11.5

Table S5. List of compounds found in the water extract of *GG (GGa)*

				[M-H]⁻			
No	Compounds	Molecular Formula	Rt (min)	Theoretical	Experimental	Delta (ppm)	RDBeq
				m/z	*m/z*		
1	2,3 Naphthalenedicarboxylic acid	$C_{12}H_8O_4$	0.85	215.0350	215.0325	-11.59	9.5
2	Pentahydroxyheptane acid	$C_{13}H_{24}O_{13}$	0.90	387.1144	387.1132	-3.24	2.5
3	Malic acid	$C_4H_6O_5$	1.07	133.0142	133.0145	1.68	2.5
4	Chorismic acid / Isochorismic acid	$C_{10}H_{10}O_6$	4.69	225.0409	225.0399	-2.67	6.5
5	p-Hydroxybenzylmalonic acid	$C_{10}H_{10}O_5$	5.43	209.0455	209.0452	-1.66	6.5
6	Liquiritin apioside	$C_{26}H_{30}O_{13}$	6.64	549.1614	549.1593	-3.76	12.5
7	Naringenin	$C_{15}H_{12}O_5$	7.78	271.0612	271.0604	-2.85	10.5
8	Liquiritigenin	$C_{15}H_{12}O_4$	8.56	255.0663	255.0655	-3.11	10.5
9	Licorice saponin G2	$C_{42}H_{62}O_{17}$	8.99	837.3914	837.3870	-5.26	12.5
10	Echinatin	$C_{16}H_{14}O_4$	9.34	269.0819	269.0811	-2.91	10.5
11	Glycyrrhizic acid	$C_{42}H_{62}O_{16}$	9.90	821.3965	821.3929	-4.42	21.5
12	Licorice saponin B2	$C_{42}H_{64}O_{15}$	10.33	807.4172	807.4135	-4.70	11.5
13	Licorice saponin K2/H2	$C_{42}H_{62}O_{16}$	10.37	821.3954	821.3926	-4.70	12.5
14	Formononetin	$C_{16}H_{12}O_4$	10.38	267.0663	267.0655	-2.97	11.5
15	Glycybridin J	$C_{21}H_{22}O_6$	10.69	369.1344	369.1331	-3.28	11.5
16	Licoflavone C	$C_{20}H_{18}O_5$	11.44	337.1082	337.1070	-2.96	12.5
17	Erybacin B	$C_{19}H_{18}O_5$	12.35	325.1082	325.1073	-2.51	11.5
18	Kanzonol A	$C_{20}H_{20}O_5$	12.54	339.1238	339.1228	-2.85	11.5
19	Glabridin	$C_{20}H_{20}O_4$	12.93	323.1289	323.1280	-2.67	11.5
20	Glabrone or Kanzonol W	$C_{20}H_{16}O_5$	12.94	335.0925	335.0915	-3.00	13.5
21	Glabrene	$C_{20}H_{18}O_4$	13.11	321.1132	321.1122	-3.09	12.5
22	Kanzonol Y	$C_{25}H_{30}O_5$	13.40	409.2021	409.2006	-3.56	11.5
23	Glycybridin C	$C_{25}H_{30}O_5$	13.83	409.2021	409.2008	-3.02	11.5
24	Phaseollin	$C_{40}H_{36}O_8$	14.56	643.2337	643.2338	0.03	23.5
25	3-Hydroxyglabrol	$C_{25}H_{28}O_5$	15.20	407.1864	407.1859	-1.27	12.5
26	Glabrol	$C_{25}H_{28}O_4$	15.70	391.1915	391.1914	-0.26	12.5

Table S6. List of compounds found in the water/methanol extract of GG (GGb)

No	Compounds	Molecular Formula	[M-H]$^-$ Rt (min)	Theoretical m/z	Experimental m/z	Delta (ppm)	RDBeq
1	Pentahydroxyheptane acid	$C_{13}H_{24}O_{13}$	0.91	387.1144	387.1130	-3.63	2.5
2	Malic acid	$C_4H_6O_5$	1.03	133.0142	133.0144	1.03	2.5
3	p-Hydroxybenzylmalonic acid	$C_{10}H_{10}O_5$	5.45	209.0455	209.0452	-1.47	6.5
4	Liquiritin apioside	$C_{26}H_{30}O_{13}$	6.63	549.1614	549.1591	-4.09	12.5
5	Neoliquiritin 2''-apioside	$C_{26}H_{30}O_{13}$	7.55	549.1614	549.1589	-4.54	12.5
6	Isoliquiritin apioside	$C_{26}H_{30}O_{13}$	7.64	549.1614	549.1588	-4.65	12.5
7	7,5′-dihydroxy-6,3′-dimethoxy-isoflavone-7-*O*-β-d-glucopyranoside	$C_{23}H_{24}O_{11}$	7.93	475.1232	475.1226	-1.99	12.5
8	Naringenin	$C_{15}H_{12}O_5$	8.01	271.0612	271.0603	-3.20	10.5
9	Liquiritigenin	$C_{15}H_{12}O_4$	8.67	255.0663	255.0657	-2.44	10.5
10	Licorice saponin G2	$C_{42}H_{62}O_{17}$	9.00	837.3914	837.386	-6.42	12.5
11	Echinatin	$C_{16}H_{14}O_4$	9.33	269.0819	269.0812	-2.57	10.5
12	Glycyrrhizic acid	$C_{42}H_{62}O_{16}$	9.92	821.3965	821.3926	-4.78	12.5
13	Licorice saponin B2	$C_{42}H_{64}O_{15}$	10.29	807.4172	807.4133	-4.85	11.5
14	Licorice saponin K2/H2	$C_{42}H_{62}O_{16}$	10.36	821.3954	821.3926	-4.71	12.5
15	Formononetin	$C_{16}H_{12}O_4$	10.39	267.0663	267.0655	-3.08	11.5
16	Licoflavone C	$C_{20}H_{18}O_5$	11.05	337.1082	337.1071	-3.14	12.5
17	Erybacin B	$C_{19}H_{18}O_5$	12.35	325.1082	325.1072	-2.79	11.5
18	Kanzonol A	$C_{20}H_{20}O_5$	12.52	339.1238	339.1228	-2.85	11.5
19	Glabridin	$C_{20}H_{20}O_4$	12.93	323.1289	323.1283	-1.71	11.5
20	Glabrone or Kanzonol W	$C_{20}H_{16}O_5$	12.96	335.0925	335.0916	-2.56	13.5
21	Glabrene	$C_{20}H_{18}O_4$	13.09	321.1132	321.1124	-2.69	12.5
22	Kanzonol Y	$C_{25}H_{30}O_5$	13.40	409.2021	409.2011	-2.36	11.5
23	Glycybridin C	$C_{25}H_{30}O_5$	13.84	409.2021	409.2011	-2.22	11.5
24	Phaseollin	$C_{40}H_{36}O_8$	14.90	643.2337	643.2324	-2.05	23.5
25	3-Hydroxyglabrol	$C_{25}H_{28}O_5$	15.21	407.1864	407.1858	-1.42	12.5
26	Glabrol	$C_{25}H_{28}O_4$	15.69	391.1915	391.1913	-0.52	12.5

Table S7. Fatty acid methyl esters composition of the seed oil extract of EP.

Peak	Rt (min)	Relative percentage (%)	Molecular Formula	Compound Name
**1**	26.37	8.75	$C_{17}H_{34}O_2$	Palmitic acid methyl ester
**2**	30.93	9.93	$C_{19}H_{32}O_2$	γ-Linolenic acid methyl ester
**3**	31.74	68.94	$C_{19}H_{34}O_2$	Linoleic acid methyl ester
**4**	31.90	8.15	$C_{19}H_{36}O_2$	9-Octadecenoic acid, methyl ester, (E)-/oleic acid methyl ester
**5**	31.98	0.72	$C_{19}H_{36}O_2$	9-Octadecenoic acid, methyl ester, (Z)-/oleic acid methyl ester
**6**	32.69	2.84	$C_{19}H_{38}O_2$	Stearic acid methyl ester
**7**	39.37	0.67	$C_{21}H_{40}O_2$	cis-13-Eicosenoic acid methyl ester
